# Retraction: Chitosan-Coated Titanium Dioxide-Embedded Paclitaxel Nanoparticles Enhance Anti-Tumor Efficacy Against Osteosarcoma

**DOI:** 10.3389/fonc.2021.712966

**Published:** 2021-06-10

**Authors:** Frontiers Editorial Office

**Affiliations:** Frontiers Media SA, Lausanne, Switzerland

The Journal and Authors retract the 9 September 2020 article cited above for the following reasons provided by the Authors:

Following publication, concerns were raised regarding image manipulation. The authors failed to provide a satisfactory explanation during the investigation, which was conducted in accordance with Frontiers’ policies.

This retraction was approved by the Chief Editors of Frontiers in Oncology and Chief Executive Editor. The authors agree to this retraction.

